# Anti-proliferative effects of a polyherbal formulation on HL-60, HCT-116, and HeLa cell lines: a docking simulation and response surface design-aided study

**DOI:** 10.3389/fchem.2025.1487887

**Published:** 2025-02-13

**Authors:** Chukwuebuka Egbuna, Kingsley C. Patrick-Iwuanyanwu, Eugene N. Onyeike, Johra Khan, Sahar AlDosari, Sadaf Jahan, Kamoru A. Adedokun, Sikiru O. Imodoye, Ibrahim O. Bello, Chukwuemelie Z. Uche, Sana Noreen, Jonathan C. Ifemeje

**Affiliations:** ^1^ African Centre of Excellence in Public Health and Toxicological Research (ACE-PUTOR), University of Port-Harcourt, Port Harcourt, Rivers, Nigeria; ^2^ Department of Biochemistry, Faculty of Science, University of Port Harcourt, Port Harcourt, Rivers, Nigeria; ^3^ Department of Medical Laboratory Sciences, College of Applied Medical Sciences, Majmaah University, Majmaah, Saudi Arabia; ^4^ Health and Basic Sciences Research Center, Majmaah University, Majmaah, Saudi Arabia; ^5^ Department of Immunology, Roswell Park Comprehensive Cancer Center, Buffalo, NY, United States; ^6^ Department of Oncological Sciences, Huntsman Cancer Institute, University of Utah, Salt Lake City, UT, United States; ^7^ Department of Biological Sciences, Southern Illinois University Edwardsville, Edwardsville, IL, United States; ^8^ Department of Medical Biochemistry and Molecular Biology, Faculty of Basic Medical Sciences, University of Nigeria, Enugu Campus, Enugu, Nigeria; ^9^ University Institute of Diet and Nutritional Sciences, The university of Lahore, Lahore, Pakistan; ^10^ Department of Biochemistry, Faculty of Natural Sciences, Chukwuemeka Odumegwu Ojukwu University, Uli, Nigeria

**Keywords:** polyherbal formulation, cancer drug discovery, anti-cancer plants, cell lines, cytotoxicity

## Abstract

Cancer is a complex global health challenge that requires novel and holistic approaches to treatment and prevention. Polyherbal medicines, composed of multiple plants with historical use in traditional medicine, have gained popularity due to their safety, cost-effectiveness, and accessibility. However, selecting the right plants and determining optimal combinations for enhanced biological effects remains challenging. To address this, a molecular docking study was conducted, targeting proteins implicated in cancer pathogenesis. The study identified bioactive compounds with strong binding energies, guiding the selection of polyherbal formulations for further experimentation. Using response surface methodology, various combinations of plant extracts were screened for their antioxidant properties and phytochemical content. Among the formulations tested, PHEE (Polyherbal Ethanolic Extract), comprising 70% soursop leaf, 5% jackfruit leaf, 5% orange peel, 15% citrus juice, and 5% apple fruit ethanolic extracts, exhibited the most potent biological activities, followed by SLEE (Soursop Leaf Ethanolic Extract), a 100% soursop leaf ethanolic extract. Design Expert Software predicted soursop leaf extract as a key contributor to desirable outcomes, attributed to its rich phytochemical composition. Cell-based assays revealed varying cytotoxic effects of the extracts on leukemia cells, with PHEE showing the highest potency (IC50 = 2.50 μg/mL), followed closely by SLEE (IC50 = 2.90 μg/mL). These effects are potentially due to the abundant acetogenins and flavonoids present in the extracts. However, caution is warranted regarding their cytotoxicity to normal cells. Apoptotic studies confirmed the ability of both PHEE and SLEE to induce programmed cell death, further supporting their potential as anticancer agents. This research underscores the importance of strategic plant combinations in polyherbal formulations and highlights PHEE as a promising candidate for further investigation in cancer treatment.

## 1 Introduction

Cancer is a complex and diverse group of diseases characterized by the uncontrolled growth and spread of cells within the body (Egbuna et al., 2021; Egbuna et al., 2023). This widespread and unregulated cellular growth poses a severe threat to global health, as it can affect nearly any tissue or organ, often leading to fatal outcomes. Various types of cancer exist, each named according to the specific cell type or region from which it originates. Common examples include leukemia, cervical cancer, breast cancer, lung cancer, prostate cancer, and many others. These different types of cancer can exhibit distinct behaviors, have specific risk factors, and require tailored treatment approaches (Ugai et al., 2022). The “war” against cancer gained significant momentum with the enactment of the National Cancer Act of 1971 in the United States by President Nixon which enable him to ask for an additional $100 million to initiate a vigorous effort to discover a cancer cure (Surh, 2021; Izbicka and Streeper, 2023). This legislation substantially boosted cancer research and treatment programs, marking a pivotal moment in the fight against this disease.

Globally, cancer accounts for nearly 1 in 6 deaths (WHO, 2018). In 2018 alone, cancer claimed the lives of over 9.6 million people, making it the second leading cause of death worldwide (Bray et al., 2018). Notably, the majority of cancer cases, approximately 90%–95%, are the result of genetic mutations induced by environmental factors, while only 5%–10% can be attributed to inherited genetics (Anand et al., 2008). In 2021, as the world commemorated the 50th anniversary of the National Cancer Act, it was clear that the battle against cancer has been characterized by a blend of successes and challenges (Bailar and Gornik, 1997; Dang, 2021; Surh, 2021; Izbicka and Streeper, 2023). Particularly in the field of cancer therapy, substantial progress has been made. These progress can be attributed to a combination of factors, including improved supportive care, the continuous refinement/repurposing of pharmaceuticals, and earlier methods for detecting cancer. Patients today benefit from more effective and targeted treatments, leading to improved survival rates and quality of life. However, the battle against cancer is far from over (Cutler, 2008; Bailar and Gornik, 1997). Despite the notable progress in cancer therapy, there are significant drawbacks and challenges in the field of cancer drug discovery. One prominent challenge lies in the development of new drugs. While there have been remarkable breakthroughs, the process of bringing a new cancer drug to market is time-consuming and expensive. Clinical trials, which are essential for ensuring the safety and efficacy of new treatments, can take years to complete, and many experimental drugs do not make it past this stage. Additionally, the cost of cancer drugs has become a major concern. Novel therapies often come with exorbitant price tags, making them inaccessible to a significant portion of the population, even in developed countries. This financial burden can limit patients’ access to life-saving treatments and exacerbate healthcare disparities. Furthermore, the rise in early-onset of cancers and the potential of chemotherapeutic relapse, underscores the need for a multifaceted approach to cancer prevention. Lifestyle factors, environmental influences, and genetic predispositions all play a role in cancer development, and addressing these factors presents complex challenges.

Mutation/defects in certain genes and abnormal protein functions have been linked to cancer pathogenesis and progression. For instance, the abnormal activation of PI3K/AKT, mTORC1, ERK/MAPK, STAT3/5, IDH2, Wnt/β-catenin, and NF-κB pathways are linked to the relapse and pathogenesis of cancer such as acute myeloid leukemia (Park et al., 2010; Fadeev et al., 2015; Gruszka et al., 2019; Dai et al., 2021). In other words, inhibitors or drugs that target several signaling pathways may be used to overcome treatment resistance in cancer (Park et al., 2010). Mutation in isocitrate dehydrogenases (IDHs) has been linked to cancer. IDHs are digestive enzymes that belong to the citric acid cycle. They work by catalyzing the oxidative decarboxylation of isocitrate into alpha-ketoglutarate. Isocitrate dehydrogenase mutations were initially discovered in AML in conjunction with the sequencing of the first AML genome in 2008 (Issa and DiNardo, 2021). It is currently known that IDH1 or IDH2 mutations are present in 8% and 12%, respectively, of acute myeloid leukemias (Issa and DiNardo, 2021). IDH2 mutations have also been described in various malignant cancers. The conversion of 2-hydroxyglutarate from α-ketoglutarate (α-KG) may be facilitated by IDH mutation (2-HG). High frequency mutations in IDH2 have been detected in AML, including chondrosarcoma, glioma, solid papillary carcinoma with reverse polarity and angioimmunoblastic T cell lymphoma (Yang et al., 2012; Guo et al., 2021). Mutations in IDH1/2 genes is associated with DNA hypermethylation and epigenetic dysregulation (Libura et al., 2021).

In silico screening of large databases of prospective compounds/inhibitors may reveal compounds with a high possibility of binding to a target protein in cancer research (Shaikh et al., 2022; Rudrapal and Egbuna, 2022). The process of molecular docking creates a variety of ligand conformations and orientations that fit against the target while suggesting the best fits through scoring (Rudrapal and Egbuna, 2022). This technique can guide researchers in making decision on the type of plants for selections that contains desired bioactives. Medicinal plants (herbs) have long been explored as potential sources for developing new drugs to combat various diseases, particularly cancer (Arul et al., 2018; Saravanan et al., 2020). Several medicinal plants such as garlic (*Allium sativum*), Asian ginseng (*Panax ginseng*), turmeric (*Curcuma longa*), and green tea (*Camellia sinensis*), have demonstrated beneficial effects in both experimental and clinical studies for cancer prevention and treatment (Fani Pakdel et al., 2021; Saravanan et al., 2020). Some herb-derived compounds, such as vinca alkaloids and taxol analogs, have been used in cancer chemotherapy (Egbuna et al., 2019). This study, based on previous and present *in silico* studies (Egbuna et al., 2021; Egbuna et al., 2023), selected seven plants which contains promising bioactive compounds with the best molecular docking scores. They include apple fruit (*Malus domestica*), jackfruit leaf (*Artocarpus heterophyllus*), soursop leaf (*Annona muricata*), sweet orange fruit peel (*Citrus sinensis*), lemon fruit (*Citrus limon*), lime fruit (*Citrus aurantifolia*), and shaddock fruit (*Citrus maxima*). A study by Koseoğlu and Al-Taie (2022), explored the potential chemo-preventive roles of *M. domestica* in mitigating the risk of colorectal cancer and offers suggestive insights into its clinical application. A study by Sun et al. (2017) highlights the traditional use of *A. heterophyllus* in folk medicine and identifies artocarpin as a promising colorectal cancer chemopreventive agent, targeting Akt kinase, inducing cell cycle arrest, and showing potential in colitis-associated tumorigenesis in mice. A review by Ilango et al. (2022) discusses the growing use of *A. muricata* for cancer treatment based on its efficacy. It further stated that *A. muricata*’s compounds, including alkaloids, phenols, and acetogenins, has been explored for their potential in treating cancer by inhibiting proliferation, modulating cell death, and impacting cancer-related genes and proteins, making them promising anti-cancer agents. Nair et al. (2018) reports that citrus peels, often discarded, possess untapped potential as anticancer agents. It further stated that various citrus species, including *C. sinensis, C. maxima, C. limon* and *C. reticulata*, demonstrated significant activity against cancer cells *in vitro* and *in vivo*, making them promising candidates for cancer prevention and treatment. Also, in a systematic review and meta-analysis conducted by Cirmi et al. (2018), they found that individuals with the highest Citrus fruit intake had a 50% lower risk of oral cavity and pharyngeal cancer, based on 17 included studies.

Given the multifaceted nature of life-threatening diseases like cancer, a combination of anti-cancer herbs through polyherbal formulation might offer more promising results than single-herb therapies. Polyherbalism is a treatment strategy that combines a variety of herbs or botanical medicines to address health issues or enhance wellbeing (Parasuraman et al., 2014; Karole et al., 2019; Govindaram et al., 2022; Punia et al., 2023). Traditional therapeutic systems all around the world, including Ayurveda (Karole et al., 2019), Traditional Chinese Medicine (TCM), and Native American medicine, have included this practise for centuries. Polyherbalism provides advantages unattainable with single herbs, yielding high efficacy even at safe high doses (Karole et al., 2019). Synergism operates through pharmacokinetic (enhanced absorption, distribution, metabolism, elimination) and pharmacodynamic (diverse mechanisms targeting similar therapeutic activity) interactions, enhancing therapeutic effects in various diseases (Karole et al., 2019). This study aims to evaluate the polyherbal effects of apple fruit, jackfruit leaf, soursop leaf, sweet orange fruit peel, lemon fruit, lime fruit, and shaddock fruit ethanolic extracts on cancer cell lines to explore their potential ameliorative effects on cancer treatment. This approach reflects a growing interest in holistic and multi-component treatments for complex diseases.

## 2 Materials and methods

### 2.1 *In silico* study

#### 2.1.1 Ligand curation and preparation

A literature search was done to identify phytochemicals previously reported to have anti-cancer properties. Phytochemistry: An *in silico* and *in vitro* Update edited by Kumar and Egbuna (2018), Phytochemicals as Lead Compounds for New Drug Discovery edited by Egbuna et al. (2019), and Drug Development for Cancer and Diabetes: A Path to 2030 edited by Saravanan et al. (2020) were some of the sources consulted. The chemical structures (in 3D SDF) of 313 compounds, as well as conventional anticancer medicines with their associated CIDs, were retrieved from the NCBI PubChem database. VConf software (VeraChem LLC) was used to convert 2D SDF files to 3D SDF formats, while ChemDraw Ultra 12.0 was used to draw chemicals not found in the PubChem and ChemSpider databases (CambridgeSoft). To make it easier to integrate into PyRx programme, all of the produced ligands were compressed into a single SDF file using Open Babel software (openbabel.org).

#### 2.1.2 Protein preparation

The IDH2 protein (with PDB ID 5SVN and 2.10 Å resolution) was obtained in.pdb format from the Protein Data Bank (https://www.rcsb.org/) and processed using BIOVIA Discovery Studio Visualizer 2021 v21.1.0.20298 (Dassault Systèmes: https://www.3ds.com) according to Qasaymeh et al. (2019). The active molecules identified in association with the protein served as a model for predicting the active site of the protein. The generated model is then validated via comparison with experimental data. Water molecules and hetero atoms were removed during processing, whereas polar hydrogens were added.

#### 2.1.3 Active site prediction

The PDB, PrankWeb (P2Rank), Biovia Discovery Studio, and scholarly materials were used to determine the active sites of the proteins. PrankWeb provided a forecast along with its expected center coordinates. PrankWeb is a cutting-edge machine learning-based platform for online protein ligand binding site prediction (https://prankweb.cz/). The forecasts from the sources cited above were reconciled. For this investigation, the amino acid residues that seem to be common to them were chosen (Table 1). To guarantee that the target protein binding site is covered by the grid box configuration in the PyRx programme, the correct predicted amino acid residue was chosen.

**TABLE 1:** Coordinates of autogrid (docking box) on the active sites of proteins for docking.

S/No	Protein	Resolved center coordinate	Dimensions in Angstrom	Targeted amino acid residue on active site
1	IDH2	X −14.4960. Y: 10.8503, Z: −29.0738	X: 25.0000, Y: 25.0000, Z: 28.8688	115, 117, 122, 149, 299, 348, 367

#### 2.1.4 Molecular docking studies

According to Dallakyan and Olson’s (2015) method, molecular docking simulations were carried out using PyRx software version 0.8 (https://pyrx.sourceforge.io) (2015). PyRx is a program for high-throughput virtual screening of compounds against protein targets using molecular docking simulations. By analyzing the binding energy of compounds in kcal/mol, it was possible to determine which substances have the highest chances of forming a strong bond with a protein. In this work, the 3D SDF-formatted ligands that had been generated and compressed were loaded into PyRx using the built-in OpenBabel graphical user interface. The conjugate gradient approach was used to minimize energy using the Universal Force Field (UFF), with a total number of steps set at 200. The update’s step count was set to 1 and then 0.1. Afterwards, all ligands were converted into AutoDock ligands to reduce energy use (pdbqt). Docking simulation was run with an exhaustiveness level of 8. The ligand with the greatest binding affinity was identified as having the highest binding energy (most negative) (Prasanth et al., 2020). Using BIOVIA Discovery Studio, specific interactions of the optimum docking positions were shown. Docking procedure validation was done.

### 2.2 Collection of plant materials

The result obtained from the *in silico* study informed the choice of seven plant samples listed below for polyherbal formulation. The leaves of soursop and jackfruit were harvested from the researcher’s family compound in Nkwelle Ogidi, Anambra State, Nigeria while apple fruit, lime orange, sweet orange, lemon, and shaddock fruits were purchased from Ose Main Market Onitsha, Anambra State, Nigeria. Sweet orange peels were freely obtained from a fruit seller in Afor Igwe Market Ogidi, Anambra State, Nigeria. All samples were collected between the months of March and April 2021. The samples were identified by a plants Taxonomist.

It is important to note that phytochemical variability in plant and fruit samples—arising from factors such as geographic origin, cultivation methods, and harvest times—could influence the consistency and reproducibility of results. Such variability may limit the generalization of the findings from this study. Therefore, future research should consider standardizing these variables to ensure consistency in the phytochemical profiles of the plant materials used in formulations.

**Table:** 

Name	Scientific name	Part used
Apple	*Malus domestica*	Fruit
Jackfruit	*Artocarpus heterophyllus*	Leaf
Soursop	*Annona muricata*	Leaf
Sweet orange	*Citrus sinensis*	Peel and Whole fruit
Lemon	*Citrus limon*	Whole fruit
Lime	*Citrus aurantifolia*	Whole fruit
Shaddock	*Citrus maxima*	Whole fruit

### 2.3 Sample extraction and preparation

The samples collected were washed with tap water, followed by distilled water, to remove dirt and debris. The apple fruits were sliced into pieces, shade-dried, and ground into a fine powder. Similarly, the sweet orange peels and the leaves of soursop and jackfruit were shade-dried and ground into a fine powder.

For extraction, 500 g of each powdered sample was soaked in 2 L of 80% ethanol for 3 days. The mixtures were filtered separately using sterile Whatman No. 1 filter paper, and the filtrates were concentrated using a rotary evaporator. The resulting crude extracts were stored in a refrigerator at 4°C until analysis.

Fresh juices of sweet orange, lemon, lime, and shaddock were obtained from 1 kg of each fruit. The juices were concentrated to a slurry using a rotary evaporator. Desired quantities of the concentrated slurry, expressed as percentages, were used in the polyherbal formulation using design expert software layout. Additionally, the fresh juices were extracted with 80% ethanol, combined, filtered, and concentrated using a rotary evaporator. The crude extracts were stored in a refrigerator at 4°C until analysis.

### 2.4 Polyherbal formulation

To ascertain the best ratio to mix the plant extracts for the polyherbal formulation, Design Expert 7.0.0 software was used to design the experiment. The plant extracts were mixed in various proportions of 26 formulations including 5 replica as generated by Design Expert software. Because all of the components have the same range of 0–100 and there were no constraints on the design space, the simplex-centroid mixture design was chosen for the experiment (Rahim et al., 2016).

### 2.5 Determination of DPPH, total phenolic content and total flavonoid content of extracts

#### 2.5.1 DPPH radical scavenging assay

DPPH (2,2-diphenyl-1-picryl-hydrazyl-hydrate) assay was carried out as described by Ahmed and Iqbal (2018). The ability of extracts to scavenge 2, 2-diphenyl l-picrylhydrazyl stable radicals was used to determine their antioxidant activity.

##### 2.5.1.1 Principle

The DPPH technique was based on reducing DPPH in the presence of antioxidant capable of donating hydrogen. The ability of the extracts to donate hydrogen reduces the colour of DPPH. DPPH is one of the chemicals that contains a proton free radical with a distinct absorption that is greatly reduced when exposed to proton radical scavengers.

##### 2.5.1.2 Procedure

One mililitre of the sample was put in an Eppendorf tube along with 1 mL of the DPPH solution, which was made by dissolving 0.001 mg of DPPH in 12 mL of methanol. The tube was then covered with aluminum foil for 30 min. After 30 min, the absorbance was measured in a spectrophotometer at 517 nm against a blank. The experiment was done in triplicate. The following formula was used to obtain the percent radical scavenging activity:

$$\%\;Radical\;scavenging\;activity=\frac{A_{o}-A_{1}}{A_{0}}\;x\;100$$

Where, A_0_ blank = Absorbance of control.

A_1_ sample = Absorbance of test samples.

#### 2.5.2 Determination of total phenolic content (TPC)

As indicated by Oke et al. (2020), the total phenolic content of the samples was evaluated using Folin-Ciocalteu reagent and Na_2_CO_3_.

##### 2.5.2.1 Principle

The Folin–Ciocalteu reagent is reduced in the presence of phenolics (by electron transfer), resulting in the generation of molybdenum–tungsten blue in alkaline medium, which absorbance was measured spectrophotometrically at 760 nm.

##### 2.5.2.2 Procedure

One milliliter (1 mL) of the filtrate (extract) was added to a clean test tube, followed by 1 mL of Folin-Ciocalteu and 2 mL of 20% Na_2_CO_3_, and the tube was spun at 4000 rpm for 20 min. The mixture was incubated at room temperature for 2 h before being measured spectrophotometrically at 756 nm against a water blank. Gallic acid was used as a standard in water (10–50 mg/L) to create the standard curve. TPC was calculated per gram of dried sample extract and reported as mg Gallic Acid Equivalent (GAE).

#### 2.5.3 Determination of total flavonoid content (TFC)

Aluminium chloride method was used to determine the flavonoid content of the samples using rutin as standard as described by Oke et al. (2020).

##### 2.5.3.1 Principle

This method is based on the formation of flavoniod-aluminum complex. When flavones and flavonols’ C-4 keto group and either their C-3 or C-5 hydroxyl groups are combined with aluminum chloride, stable complexes with acidic functions are formed. Additionally, it combines with the ortho-dihydroxyl groups on the A and B rings of flavonoids to form molecules that are acid labile.

##### 2.5.3.2 Procedure

A test tube was filled with 1 mL (1 mL) of the sample (extract) filtrate, 3 mL of methanol, 0.2 mL of 10% aluminium chloride, and 0.2 mL of 1M potassium acetate and measured in a spectrophotometer at 756 nm. The calibration curve was generated by dissolving 1 g of Rutin in 1,000 mL and diluting it with 1,000 mg/L methanol as the stock.

### 2.6 Cell viability assay

#### 2.6.1 Determination of the effect of extracts on cell proliferation by 3-(4,5-dimethylthiazol-2-yl)-2,5-diphenyltetrazolium bromide (MTT) assay

Human leukemic cells (HL-60) (ATCC^®^) were used to study the antiproliferative effects of ethanolic extracts of apple, citrus juice, jackfruit leaf, orange peel, and soursop leaf on cell viability by the modified method described by Ansari et al. (2021).

##### 2.6.1.1 Principle

MTT is converted into a purple-coloured formazan product with a maximum absorbance near 570 nm by viable cells with an active metabolism. Because cells lose their ability to convert MTT into formazan when they die, colour formation acts as a helpful and convenient indicator of only live cells. Thus, the quantity of viable cells in culture determines how much MTT is reduced to formazan.

##### 2.6.1.2 Procedure

Twenty thousand cells/wells were seeded in 96 well plates containing Dulbecco’s modified eagle medium (DMEM); fetal bovine serum; L-glutamine; penicillin; streptomycin; selenium chloride and were kept in CO_2_ incubator at 37°C. The cells were then respectively treated with 2, 10, 20 and 40 μg/mL of ethanolic extracts of apple fruit, citrus juice, jackfruit leaf, orange peel, and soursop leaf for 48 h and were processed for the MTT assay. In the control group, extract was not added. Cells were then treated with MTT (5.0 mg/mL) for 4 h followed by treatment with 1% dimethyl sulfoxide, and finally, absorbance was read at 570 nm using enzyme-linked immunosorbent assay (ELISA) reader.

##### 2.6.1.3 Calculation

The percentage growth inhibition was calculated using the following formula,

$$\%\;Cell\;inhibition=100-[\left(A_{t}-A_{b}/\left(A_{c}-A_{b}\right)\right)]\;x\;100$$

Where, A_t_ = Absorbance of test compound, A_b_ = Absorbance of blanck and A_c_ = Absorbance of control.

#### 2.6.2 Apoptotic assay by 4′,6-diamidino-2-phenylindole (DAPI) staining

The human leukemia cancer cells (HL-60) were treated with the ethanolic extracts of apple fruit, citrus fruit juice, jackfruit leaf, orange peel and soursop leaf for 48 h and were further treated with 4% paraformaldehyde, then stained with DAPI (1.0 μg/mL) under a dark environment. DAPI stained cells were examined using fluorescence confocal scanning microscope (Zeiss, Germany).

## 3 Results

### 3.1 Molecular docking study

The results obtained from IDH2 molecular docking study is presented in Table 2. The results showed that hypericin had the best binding energy of −13.3 kcal/mol. This was followed by diosmin with the binding energy of −11.9 kcal/mol. Other top performing phytochemicals with their respective binding energies (kcal/mol) are withanolide (−11.5), montamine (−11.4), bruceatin (−11.3), rutin (−11.3), tomatidine (−11.3), baicalin (−11.2), and naringin (−11).

**TABLE 2:** Molecular docking scores of IDH2 protein inhibitors.

S/N	Compound	PubChem ID	Binding energy (kcal/mol)	Sources (Kumar and Egbuna, 2018; Egbuna et al., 2019; Saravanan et al., 2020)
1	Hypericin	3,663	−13.3	Genera *Hypericum* (Saint John’s wort)
2	Diosmin	5,281,613	−11.9	Citrus fruits (oranges and lemons) and peel extracts, hyssop, figwort
3	Withanolide	53,477,765	−11.5	Nightshade plant family, e.g., *Datura, Solanum, Withania, Jaborosa*
4	Montamine	160,679,561	−11.4	Seeds of *Centaurea Montana*
5	Bruceantin	5,281,304	−11.3	*Brucea antidysenterica*
6	Rutin	5,280,805	−11.3	Citrus leaves (orange and lime), tomato, green tea, fenugreek, olive
7	Tomatidine	65,576	−11.3	Stems and leaves of tomato plants, and in the fruits at low concentrations
8	Baicalin	64,982	−11.2	Plants in Genus *Scutellaria* and in *Oroxylum indicum*
9	Naringin	442,428	−11.0	Citrus fruits, especially in grapefruit
10	Polyphyllin	72,960,700	−11.0	*Paris polyphylla*
11	Neohesperidin	442,439	−10.9	Citrus fruits (e.g., oranges and lemons), peel extracts and inedible ones
12	Vicenin-2	442,664	−10.8	Sweet oranges, *Ocimum sanctum,* buckwheats, fenugreeks
13	Silymarin	5,213	−10.7	Seeds of milk thistle *Silybum marianum* (L.)
14	Solanine	262,500	−10.7	Nightshade family, e.g., genus Solanum, e.g., potato, tomato, eggplants
15	Glycyrrhizic acid	14,982	−10.6	Licorice (Root extract), Glycyrrhiza glabra (Fabaceae)
16	Epigallocatechin galate	65,064	−10.6	Green, white and black tea. Trace in apple skin, plums, onions, hazelnuts
17	Laricitin	102,401,707	−10.6	*Vitis vinifera* red grape
18	Verbascoside	5,281,800	−10.6	Plants of Verbenaceae, Olive, Lamiaceae family
Standard IDH2 drug				Target
1	Enasidenib	89,683,805	−10.7	Isocitrate dehydrogenase 2 (IDH2) inhibitor
Other top performing anticancer drugs				
2	Venetoclax (ABT-199)	49,846,579	−11.9	Bcl-2 inhibitor
3	Guadecitabine	135,564,655	−11.8	DNA (cytosine-5)-methyltransferase 1 (DMNT1) inhibitor
4	Etoposide (Vepesid)	36,462	−11.2	Anticancer drug (semisynthetic derivative of podophyllotoxin)
5	Idarubicin	42,890	−10.9	Topoisomerase II poison (Prevents DNA unwinding)
6	Sorafenib	216,239	−10.9	FLT3 inhibitor
7	Daurismo/Glasdegib	25,166,913	−10.8	SMO inhibitor
8	Ivosidenib	71,657,455	−10.5	Isocitrate dehydrogenase 1 inhibitor

### 3.2 Determination of sample mixture by design expert software

In this study, Design Expert Software was used to develop experiment for polyherbal formulation with potentials of producing the best biological response (considering additive, synergistic or antagonistic effects). The Design Expert programme produced and sorted the independent variables and ‘runs’ at random. From Table 3, run 8 and its replica (run 16) which contains 100% of soursop leaf ethanolic extract produced the best responses because they had the highest DPPH, TPC and TFC. In run 8, the DPPH, TPC and TFC mean values were 60.09%, 1,097.93 μg/mL and 335.67 μg/mL respectively, while in run 16, the DPPH, TPC and TFC mean values were 65.8%, 1,112.27 μg/mL and 366.33 μg/mL respectively. The result was followed by experiment in runs 1, 4, 7, and 26. The lowest antioxidant properties was found in runs 23, 24. Run 23 contained a combination of 50% jackfruit leaf ethanolic extract and 50% apple fruit ethanolic extract with DPPH, TPC and TFC mean values of 32.94%, 225.93 μg/mL and 193.67 μg/mL respectively, while run 24 contained 100% citrus juice with DPPH, TPC and TFC mean values of 32.94%, 225.93 μg/mL and 193.67 μg/mL respectively.

**TABLE 3:** Design layout and experimental results for antioxidant properties.

			Component 1	Component 2	Component 3	Component 4	Component 5	Response 1	Response 2	Response 3
Std	Run	Space Type	A:Soursop leaf %	B:Jackfruit leaf %	C:Orange peel %	D:Citrus Juice %	E:Apple fruit %	DPPH %	TPC µg/mL	TFC µg/mL
26	20	Vertex	0	0	0	0	100	40.64	240.93	190.67
25	22	Vertex	0	0	0	100	0	35.93	259.27	208
24	17	Vertex	0	0	100	0	0	38.5	260.6	193
23	10	Vertex	0	100	0	0	0	48.15	297.6	184.33
22	8	Vertex	100	0	0	0	0	60.09	1,097.93	335.67
21	1	Center	20	20	20	20	20	60.9	550.6	257.33
20	21	AxialCB	10	10	10	10	60	38.87	185.6	189.67
19	25	AxialCB	10	10	10	60	10	53.35	286.6	173
18	6	AxialCB	10	10	60	10	10	48.72	348.6	195.33
17	19	AxialCB	10	60	10	10	10	40.32	217.93	198.67
16	5	AxialCB	60	10	10	10	10	47.87	733.93	210
15	9	CentEdge	0	0	0	50	50	33.06	228.6	187
14	12	CentEdge	0	0	50	0	50	36.2	246.93	200.33
13	14	CentEdge	0	0	50	50	0	50.8	315.27	181.33
12	23	CentEdge	0	50	0	0	50	33.7	197.6	184.33
11	3	CentEdge	0	50	0	50	0	39.42	234.6	197.67
10	26	CentEdge	0	50	50	0	0	62.13	424.27	269.33
9	13	CentEdge	50	0	0	0	50	55.05	720.27	301.33
8	11	CentEdge	50	0	0	50	0	57.42	745.27	286.33
7	7	CentEdge	50	0	50	0	0	52.8	715.6	282.33
6	4	CentEdge	50	50	0	0	0	56.27	717.27	293
5	15	Vertex	0	0	0	0	100	36.83	182.27	170.33
4	24	Vertex	0	0	0	100	0	32.94	225.93	193.67
3	18	Vertex	0	0	100	0	0	37.96	289.93	217.33
2	2	Vertex	0	100	0	0	0	38.42	194.6	193
1	16	Vertex	100	0	0	0	0	65.8	1,112.27	366.33

### 3.3 Design-expert software optimized recommended formulation

**FIGURE 1:**
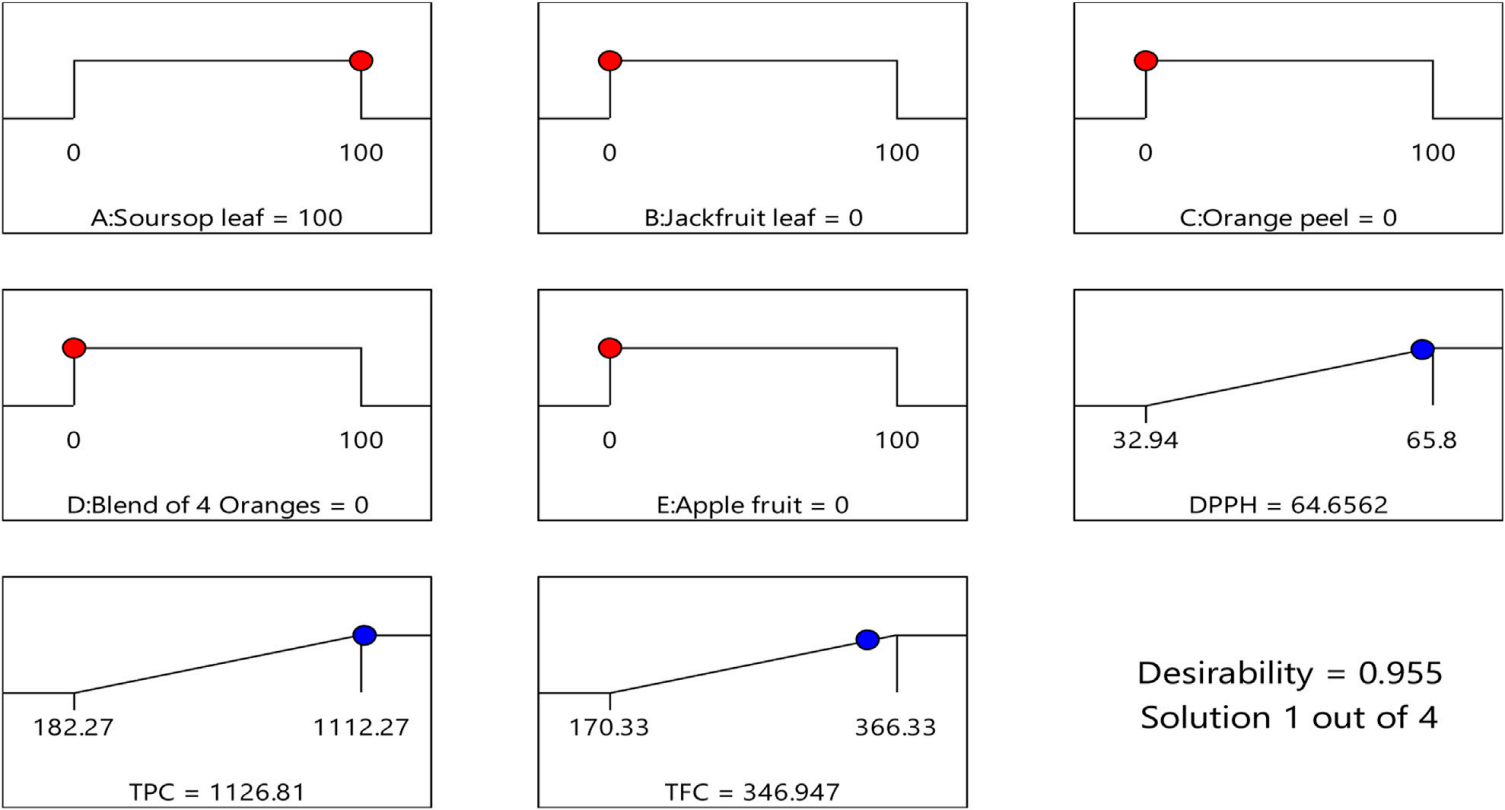
Desirability ramp for numerical optimization of the best formulation/sample that has the best DPPH, TPC and TFC values.

**FIGURE 2:**
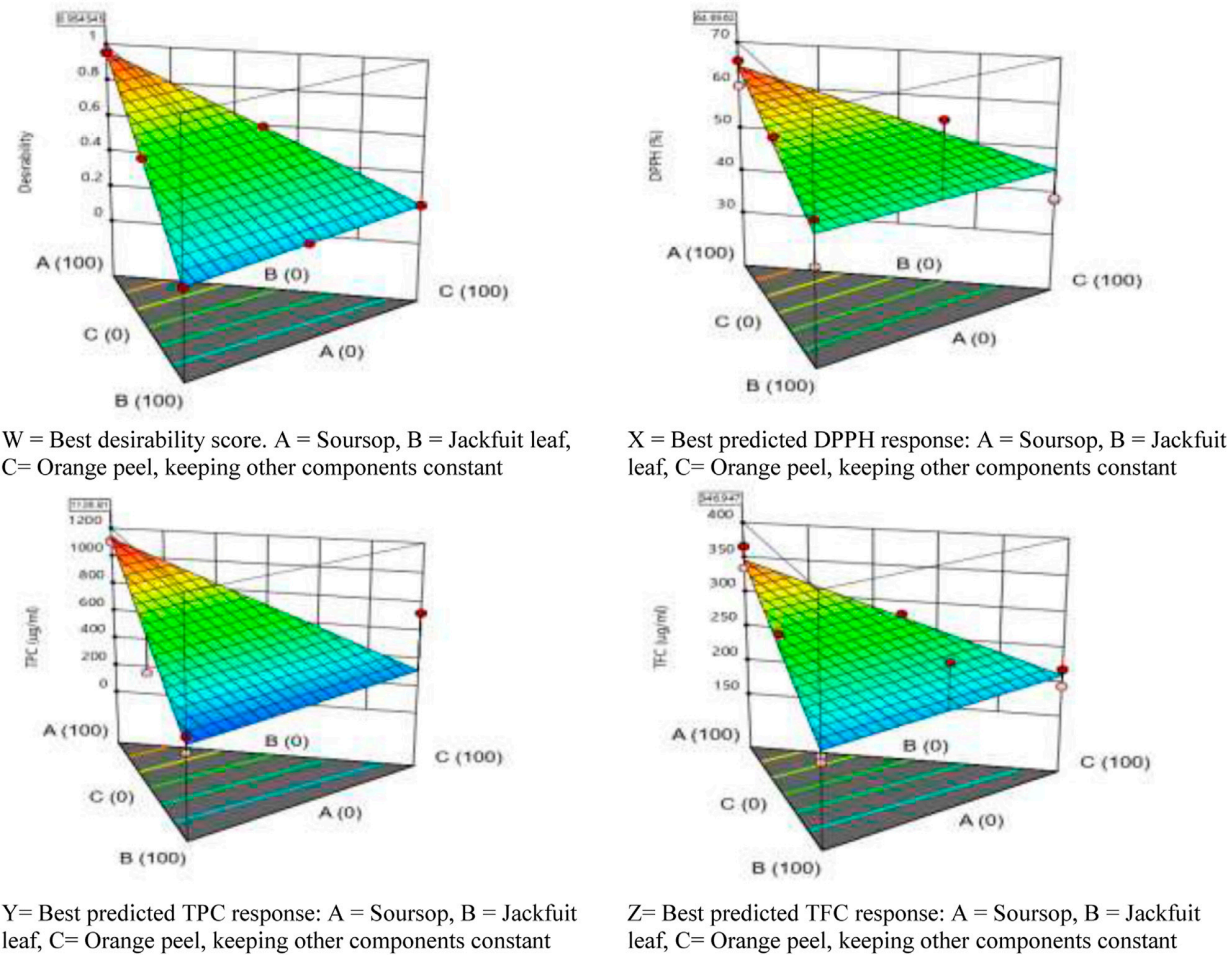
Graphical presentation of the predictable formulation that will produce the best response.

Design expert software helps in the recommendation of the best mixtures or formulations that will produce the best biological response by the exclusion or reduction of certain components that will cause antagonism. In Table 4, design expert software predicted four best formulations that will produce the best responses. In the first prediction, it suggested that the sample tube that will contain 100% soursop ethanolic leaf extract will most likely produce the best result (64.656% DPPH, 1,126.808 μg/mL TPC and 346.947 μg/mL TFC), with a desirability score of 0.955 which is close to the perfect score of 1.000 (Figures 1, 2W). From the study, two formulation were arrived at:

**TABLE 4:** Four recommended solutions for polyherbal formulation from Design Expert Software.

Number	Soursop leaf	Jackfruit leaf	Orange peel	Citrus juice	Apple fruit	DPPH (%)	TPC (µg/mL)	TFC (µg/mL)	Desirability	Desirability (w/o Intervals)	
1	100.000	0.000	0.000	0.000	0.000	64.656	1,126.808	346.947	0.894	0.955	Suggested
2	79.998	0.001	0.000	20.001	0.000	59.676	951.061	315.334	0.751	0.792	
3	78.186	0.000	0.013	21.802	0.000	59.225	935.143	312.471	0.737	0.777	
4	50.000	0.000	50.000	0.000	0.000	54.854	718.391	277.973	0.569	0.595	

PHEE (Polyherbal ethanolic extract): Mixture of 70% soursop leaf, 5% jackfruit leaf, 5% orange peel, 15% citrus juice and 5% apple fruit ethanolic extracts.

SLEE (Soursop leaf ethanolic extract): 100% Soursop leaf ethanolic extract.

In the second prediction from the Design Expert Software, it suggested that the sample tube that would contain 79.998% soursop, 0.001% jackfruit leaf and 20.001% citrus juice ethanolic extracts would produce a desirability score of 0.792 with 59.676% DPPH, 951.061 μg/mL TPC and 315.334 μg/mL TFC. In the third prediction, design expert software suggested that sample tube that would contain 78.186% soursop, 0.013% orange peel and 21.802 citrus juice would produce a desirability score of 0.777 with 59.225% DPPH, 935.143 μg/mL TPC and 312.471 μg/mL TFC. In the fourth prediction, 50.000% soursop and 50.000% orange peel will likely produce a desirability score of 0.595 with 54.854% DPPH, 718.391 μg/mL TPC and 277.973 μg/mL TFC.

Figure 2X–Z show that using soursop alone while keeping other components constant, it would likely produce better response of DPPH, TPC and TFC.

### 3.4 Cell viability studies

#### 3.4.1 Cell viability study for individual sample extracts on cell lines

**FIGURE 3:**
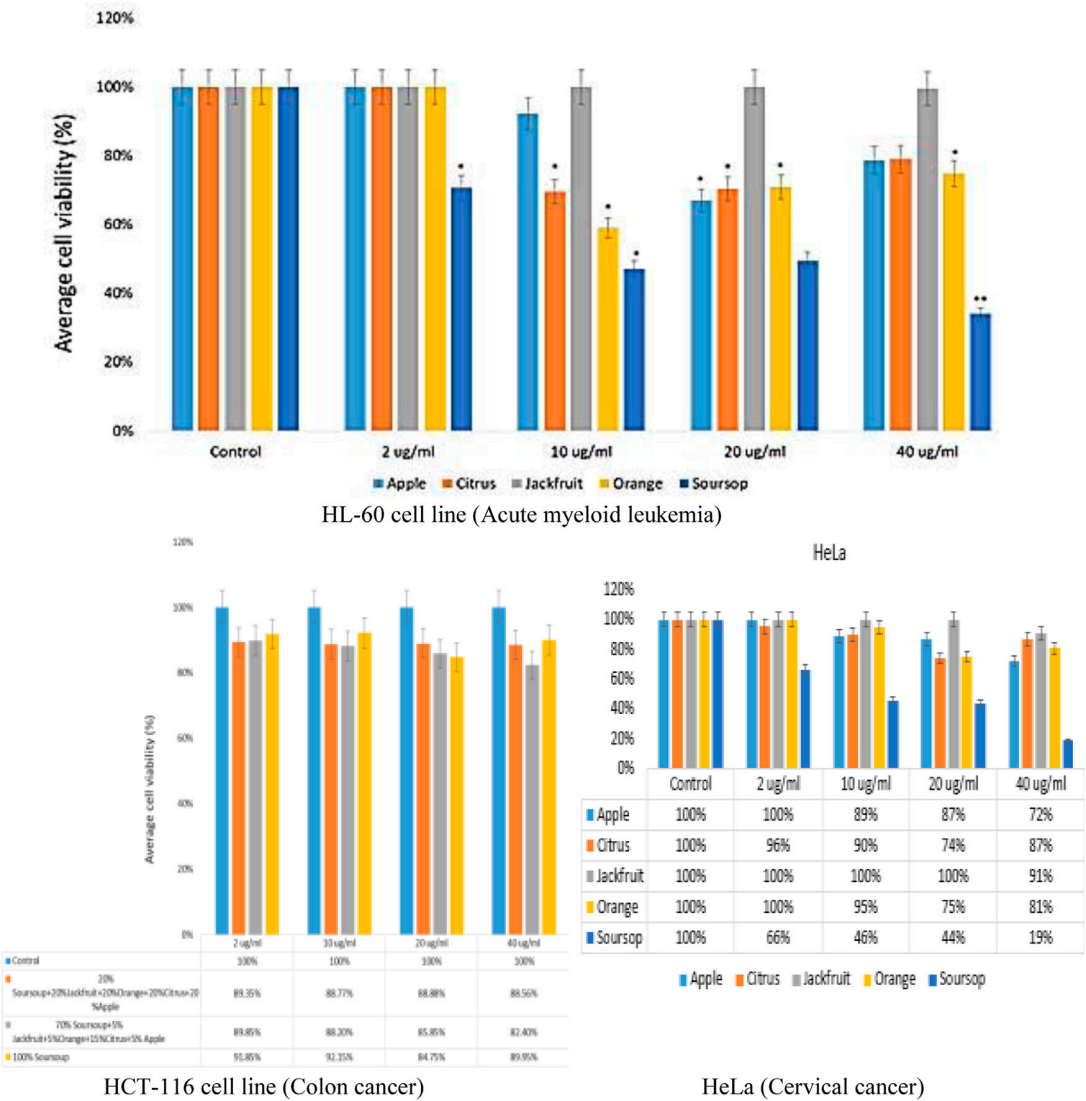
Impact of ethanolic extracts of apple fruit, citrus fruit juice, jackfruit leaf, orange peel and soursop leaf treatment on HL-60 cell viability assay by MTT assay after 48 h *p < 0.05, **p < 0.001.

The efficacy of the ethanolic extracts: apple fruit, citrus juice, jackfruit leaf, orange peel and soursop leaf on cell viability was established by MTT assay. The result showed that there was a decrease in a dose-dependent manner of cell viability after the treatments with the extracts at 2, 10, 20 and 40 μg/mL extracts (Figure 3). The result showed that the sample containing apple ethanolic extracts at concentrations of 10, 20 and 40 μg/mL caused a 92%, 67%, and 79% reduction in viable cells respectively (Figure 3). There was no reduction (100%) as found when 2 μg/mL apple extract was added to HL-60 cells. Similar observation was found for citrus juice, jackfruit leaf and orange ethanolic extracts at 2 μg/mL concentration except for soursop. In sample containing citrus fruit juice, there was 70%, 70% and 79% decrease in viable cells at concentrations of 10, 20 and 40 μg/mL while in sample containing jackfruit leaf extract only slight decrease was found at 40 μg/mL.

For orange peel extract, there was significant reduction in viable cells (59%, 71% and 75%) at concentrations of 10, 20 and 40 μg/mL respectively. The soursop leaf ethanolic extract caused 47%, 49%, and 34% reduction in viable cells at 10, 20 and 40 μg/mL respectively. The IC_50_ of the extracts on the leukemic cells was established from the data obtained using non-linear regression method by probit transformation log. The study found that soursop leaf ethanolic extract had better IC_50_ of 2.90 μg/mL followed by the IC_50_ of orange peel ethanolic extract of 36.31 μg/mL. The IC_50_ of citrus fruit juice, apple fruit and jackfruit leaf ethanolic extracts were 40.83 μg/mL, 46.77 μg/mL and 1819 μg/mL respectively. Not part of this study (the efficacy of the extracts on colon cancer and cervical cancer was also tested using HCT-116 (ATCC^®^) and HeLa cell (ATCC^®^) lines respectively. The result showed a noteworthy reduction in dose-dependent manner of cell viability after the treatments (Figure 3).

#### 3.4.2 Cell viability study for polyherbal ethanolic extract on cell lines

**FIGURE 4:**
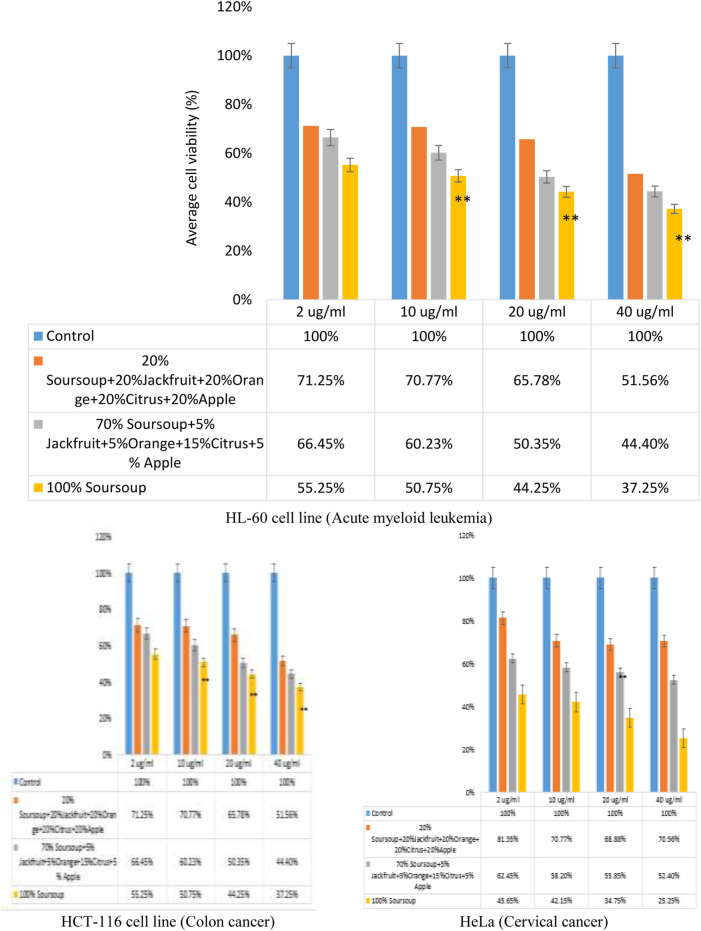
Impact of polyherbal ethanolic extracts and soursop leaf extracts treatment on HL-60 cells viability assay by MTT assay after 48 h *p < 0.05, **p < 0.001.

The efficacy of the polyherbal formulation was determined using HL-60 cell line. The result showed that there was a decrease in a dose-dependent manner of cells after the treatments with the extracts at 2, 10, 20 and 40 μg/mL extracts (Figure 4). For HL-60 cell line, the sample containing equal amount of extracts (20% soursop leaf, 20% jackfruit leaf, 20% orange peel, 20% citrus juice and 20% apple fruit ethanolic extracts) caused 71.25%, 70.77%, 65.78% and 51.56% reduction in viable cells at 2, 10, 20 and 40 μg/mL respectively. For the sample containing 70% soursop leaf, 5% jackfruit leaf, 5% orange peel, 15% citrus juice and 5% apple fruit ethanolic extracts caused a dose-dependent reduction of viable cells of 66.45%, 60.23%, 50.35% and 44.40% at 2, 10, 20 and 40 μg/mL respectively. The sample containing 100% soursop leaf extract caused 55.25%, 50.75%, 44.75% and 37.25% reduction in viable cells at 2, 10, 20 and 40 μg/mL respectively.

The IC_50_ value for the sample containing equal amount of extracts (20% soursop leaf, 20% jackfruit leaf, 20% orange peel, 20% citrus juice and 20% apple fruit ethanolic extracts) was 20.23 μg/mL. The IC_50_ value for the sample containing 70% soursop leaf, 5% jackfruit leaf, 5% orange peel, 15% citrus juice and 5% apple fruit ethanolic extracts was 14.96 μg/mL, while the IC_50_ value for the sample containing 100% soursop was 26.72%. Based on the three IC_50_ values, it can be said that the sample with the lowest IC_50_ value was a better choice hence the choice for the polyherbal formulation of 70% soursop leaf, 5% jackfruit leaf, 5% orange peel, 15% citrus juice and 5% apple fruit ethanolic extracts. The efficacy of the extracts on colon cancer and cervical cancer was also tested using HCT-116 and HeLa cell lines respectively. The result showed a noteworthy reduction in dose-dependent manner of cell viability (Figure 4).

### 3.5 Apoptotic studies

#### 3.5.1 Apoptotic study for individual sample extracts on HL-60 cell line

**PLATE 1:**
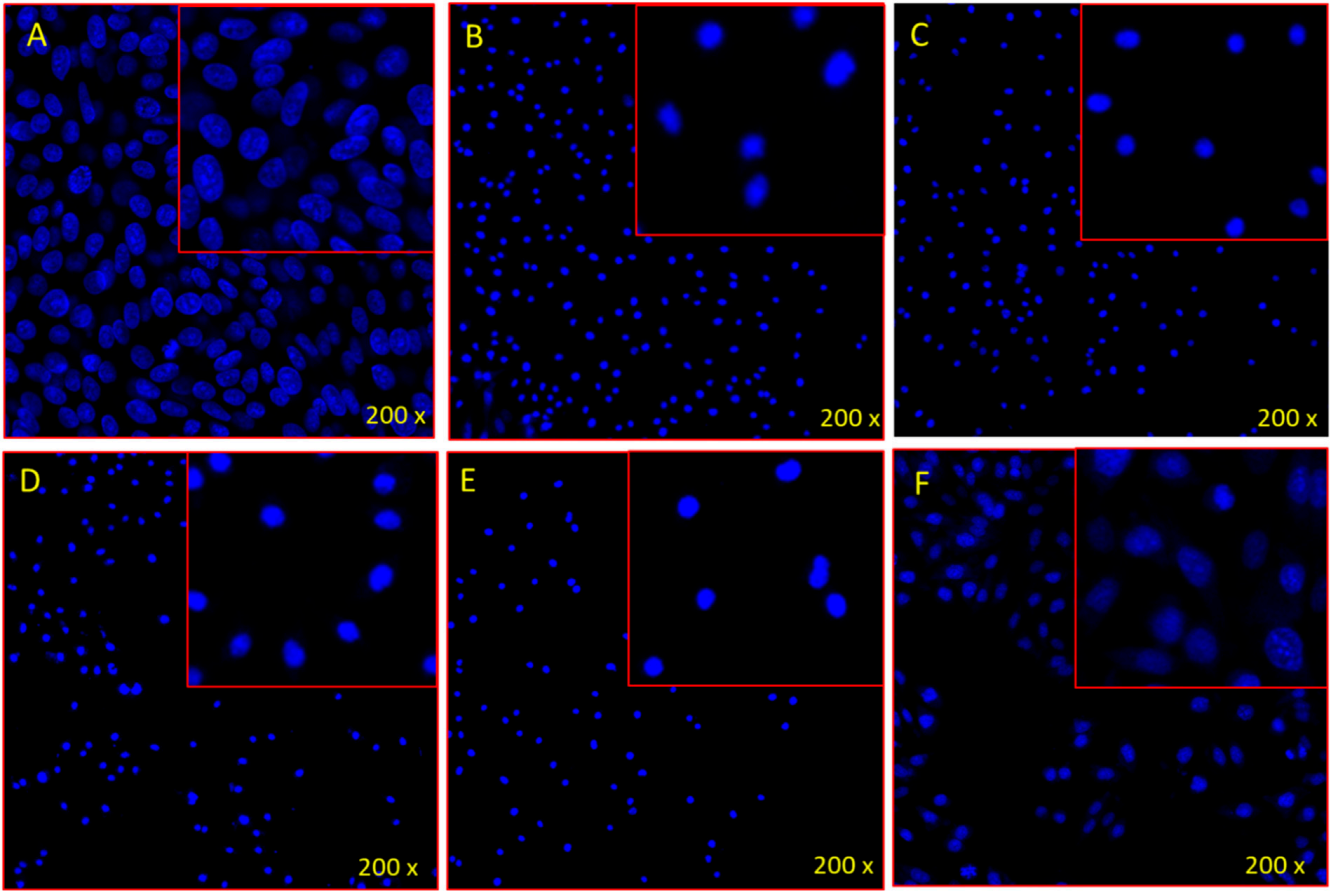
DAPI staining shows the impact of **(A)** control, **(B)** Apple fruit, **(C)** Citrus juice, **(D)** Jackfruit, **(E)** Orange peel, and **(F)** Soursop leaf on HL-60 cells stained with DAPI post 48 h treatment.

The treatment with ethanolic extracts of apple fruit, citrus fruit juice, jackfruit leaf, orange peel and soursop leaf produced substantial reduction in cancer cell proliferation. It was found that the number of DAPI stained cells were less in apple fruit, citrus juice, jackfruit, orange peel, soursop leaf extracts-treated group compared to the control cells (Plates 1A–F). The control cells (Plate 1A) showed normal and healthy cells, whereas the apple, citrus, jackfruit, orange, soursop-treated cells showed nuclear disintegration, chromatic fragmentation which were signs for the apoptosis or programmed cell death.

#### 3.5.2 Apoptotic study on formulated polyherbal ethanolic extract

**PLATE 2:**
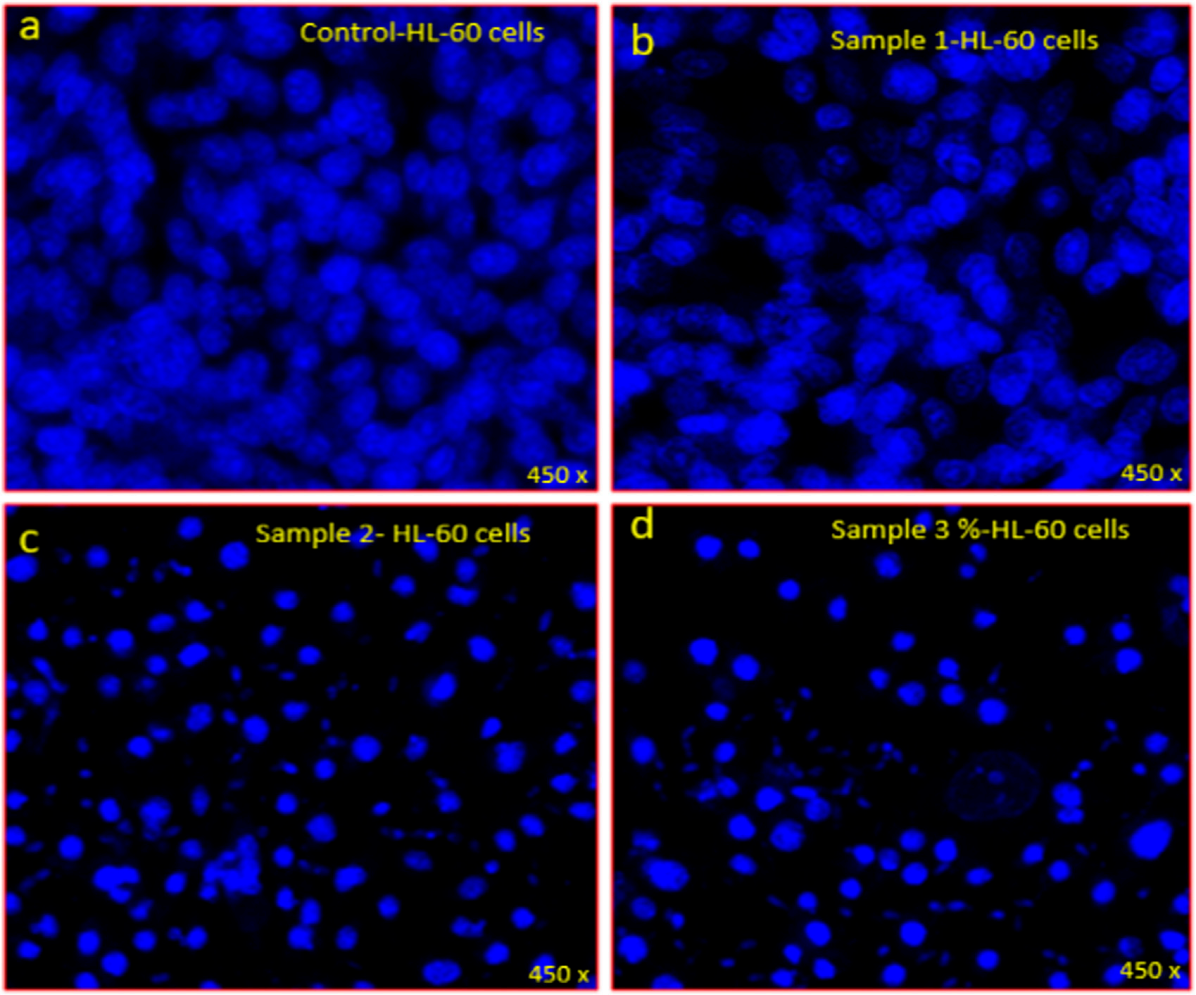
DAPI staining showing the antiproliferative effects of extracts on HL-60 leukemic cells **(A)** control, **(B)** Mixture of 20% soursop leaf, 20% jackfruit leaf, 20% orange peel, 20% citrus juice and 20% apple fruit ethanolic extracts **(C)** Mixture of 70% soursop leaf, 5% jackfruit leaf, 5% orange peel, 15% citrus juice and 5% apple fruit ethanolic extracts, **(D)** 100% Soursop leaf ethanolic extract on HL-60 cells stained with DAPI post 48 h treatment.

The nuclear and chromatin disintegration potentials on leukemic cells by the ethanolic extracts of polyherbal formulation was examined by fluorescence-activated cell sorting. The result showed no noticeable nuclear disintegration in frame A (control) and B (Mixture of 20% soursop leaf, 20% jackfruit leaf, 20% orange peel, 20% citrus juice and 20% apple fruit ethanolic extracts) (Plate 2).

In frame C (Mixture of 70% soursop leaf, 5% jackfruit leaf, 5% orange, 15% citrus juice and 5% apple fruit ethanolic extracts) and D (100% Soursop leaf ethanolic extract), there was noticeable nuclear disintegration, chromatic fragmentation which were signs of apoptosis or programmed cell death. This further confirms the choice of the two formulations:

PHEE (Polyherbal ethanolic extract): Mixture of 70% soursop leaf, 5% jackfruit leaf, 5% orange peel, 15% citrus juice and 5% apple fruit ethanolic extracts.

SLEE (Soursop leaf ethanolic extract): 100% Soursop leaf ethanolic extract.

## 4 Discussion

### 4.1 Polyherbal formulation and efficacy testing

Polyherbal medicines are made by combining many plants that have traditionally been used to cure physiological conditions, particularly by theory, error and trials founded in ancient philosophy and culture (Shan et al., 2020; Yamashiro et al., 2020; Sheth et al., 2021). Due to their high margin of safety, economic efficiency, and availability, the demand for herbal formulation utilizing traditional medicinal plants has risen (Rahim et al., 2016; Egbuna et al., 2020). However, researchers in the fields of phytochemistry, biochemistry, medicinal plant chemistry, drug discovery and development have had difficulty selecting the plants to utilize in polyherbal formulations and the proper combination that would result in larger biological effects. To circumvent this challenge, molecular docking study was first conducted using 313 curated bioactive compounds on target proteins [e.g., IDH2 protein (Table 3)] implicated in cancer pathogenesis (Egbuna et al., 2021; Egbuna et al., 2023). According to Yang et al. (2012), IDH1 and IDH2 mutations cause the generation of 2-hydroxyglutarate (2–HG) to increase while simultaneously losing their normal catalytic activity, which produces α-ketoglutarate (α-KG). Lysine histone demethylases (KDM) and the ten-eleven translocation (TET) family of DNA hydroxylases are two α-KG-dependent dioxygenases that are competitively inhibited by 2-HG, which has structural similarities with α-KG. IDH1 and IDH2 mutation-related cancers are increasingly showing abnormal histone and DNA methylation, which may affect stem cell differentiation and ultimately lead to carcinogenesis (Yang et al., 2012).

The result revealed that bioactive compounds such as hypericin had the best binding energy of −13.3 kcal/mol which was followed by diosmin (found in citrus peel) with the binding energy of −11.9 kcal/mol. Other top-performing phytochemicals with their respective binding energies (kcal/mol) are withanolide (−11.5), montamine (−11.4), bruceatin (−11.3), rutin (−11.3), tomatidine (−11.3), baicalin (−11.2), and naringin (−11). This result among other factors such as previous studies, availability of plant sample, toxicity based on ADMET study informed the choice of seven plant samples used in this study. Again, after extraction, an initial experiment of 26 runs/formulations by response surface methodology through the aid of Design Expert Software (version 7.0.0) was designed, in which different combination of the selected plant sample extracts were tested for their DPPH radical scavenging ability, total phenolic and total flavonoids contents. Because there were no restrictions on the design space and all the components had the same range of 0–100, the simplex-centroid mixture design was selected for the experiment (Rahim et al., 2016) (Table 3). In this study, a different combination of sample extracts induced different biological responses (Table 3). Some combinations produced antagonistic effects while others produced synergistic effects. The idea of synergism in polyherbal formulation was discussed by Karole et al. (2019). According to them, when numerous herbs are combined in a precise ratio for polyherbal and herbo-mineral formulations, the medicinal impact was boosted and the toxicity was reduced. They further reported that active ingredients taken from a single plant may be insufficient to provide a pharmacological effect. In other words, there was ample evidence to suggest that crude plant extracts often have higher potencies than individual ingredients.

To determine the synergistic effects of the extracts, their DPPH radical scavenging effects and the total phenolic and flavonoid contents were assessed. Based on the Design Expert Software first suggestion (Table 4; Figure 2), it predicted that a sample tube that would contain 100% soursop ethanolic leaf extract would most likely produce the best result (64.656% DPPH, 1,126.808 μg/mL TPC and 346.947 μg/mL TFC), with a desirability score of 0.955 which was close to the perfect score of 1.000 (Figures 1, 2 **W**). In the second prediction, it suggested that the sample tube that will contain 79.998% soursop, 0.001% jackfruit leaf and 20.001% citrus juice ethanolic extracts would produce a desirability score of 0.792 with 59.676% DPPH, 951.061 μg/mL TPC and 315.334 μg/mL TFC. Based on the following predictions, it can be seen that the tubes that contained a high amount of soursop produced the highest DPPH radical scavenging effect. This result was in line with several reports that have linked soursop with possessing radical scavenging effects mainly because of its unique phytochemical composition (George et al., 2015; Moghadamtousi et al., 2015; Abdul-Wahab et al., 2018; Onohuean et al., 2021; Jagtap et al., 2022; Prasad et al., 2021).

### 4.2 Cell viability and apoptotic study

Cell-based assays are often used to screen chemical collections for their impacts on cell development or for direct cytotoxic effects that eventually result in cell death (Riss et al., 2013). The MTT test, an example of a cell-based test, measures cell viability, proliferation, and cytotoxicity by measuring cellular metabolic activity. To perform this colourimetric test, metabolically active cells must convert purple formazan crystals into the yellow tetrazolium salt 3-(4,5-dimethylthiazol-2-yl)-2,5-diphenyltetrazolium bromide. The sample extracts (ethanolic extracts of apple fruit, citrus juice, jackfruit leaf, orange peel and soursop leaf) used for formulation caused a reduction in cell viability (Figures 3, 4). The IC_50_ of the extracts on the leukemic cells was established from the data obtained using a non-linear regression method by probit transformation log. The study found that soursop leaf ethanolic extract had a better IC_50_ of 2.90 μg/mL followed by the IC_50_ of orange peel ethanolic extract of 36.31 μg/mL. The IC_50_ of citrus fruit juice, apple fruit and jackfruit leaf ethanolic extracts were 40.83 μg/mL, 46.77 μg/mL and 1819 μg/mL respectively. In IC_50_ determination, an IC_50_ can be classified as having strong cytotoxicity (when less than 100 μg/mL), moderate cytotoxicity (between 101 and 200 μg/mL), or weak cytotoxicity (when more than 201 μg/mL) (Subarnas et al., 2012; Hadisaputri et al., 2021). Based on this classification, it can be stated that the ethanolic extract of soursop leaf had a very high toxic value compared to other samples, with an IC_50_ value of 2.90 μg/mL. Though the IC_50_ of orange peel ethanolic extract (36.31 μg/mL), citrus fruit juice (40.83 μg/mL), and apple fruit (46.77 μg/mL) were strong, the IC_50_ of jackfruit leaf ethanolic extracts has weak 1819 μg/mL. The strong IC_50_ values may be attributed to the sample’s phytochemical components, particularly acetogenin in soursop and flavonoids, which are both cytotoxic compounds (Yang et al., 2015; Syed Najmuddin et al., 2016). The phytochemical components of the extracts include phenols, saponins, tannins/polyphenols, and flavonoids.

The result further demonstrated the efficacy of soursop leaf ethanolic extracts as suggested by the Design Expert Software experiment. From the IC_50_ result, it can be seen that as little as 2.90 μg/mL soursop leaf ethanolic extract would be needed to cause half maximum inhibitory activity on HL-60 leukaemia cells. This was a positive attribute of soursop leaf ethanolic extract but the use must be with caution as high concentration may be cytotoxic to normal cells. This result was similar to the findings of Hadisaputri et al. (2021) who observed that ethyl acetate fraction and n-hexane fraction of soursop leaf extract exhibited an IC_50_ of 2.86 and 3.08 μg/mL on MCF7 breast cancer cell line respectively. Also, Kuete et al. (2016) found that the IC_50_ values for soursop leaf were less than 1 μg/mL for CCRF-CEM cells and less than 10 μg/mL for its MDR subline CEM/ADR5000 cells. In another experiment by Pieme et al. (2014), they found that all of the examined extracts for different parts of soursop ethanolic extract reduced the number of HL-60 cells that proliferated in a concentration-dependent manner by stopping cell growth, with an IC_50_ ranging from 6–49 ug/mL. They proposed that this could have been possible by the disruption of MMP (mitochondrial membrane permeabilization), the production of reactive oxygen species (ROS), and the arrest of the G0/G1 cell cycle.

Furthermore, the apoptotic study in this work revealed that soursop leaf ethanolic extract (solely or in combination with other extracts up to 70%) caused apoptosis in the HL-60 cell line which was visible under fluorescence confocal scanning microscope (Plates 1, 2). This was a positive attribute as it could be used to treat cancer. This result was in agreement with the findings of Pieme et al. (2014) who under a fluorescence microscope observed that soursop-treated HL-60 cells underwent apoptosis through cell clearance. The possible apoptotic induction mechanism behind this according to Alali et al. (1999), was that soursop acetogenin prevents the mitochondria of cancer cells from producing adenosine triphosphate (ATP), interrupting the energy supply and making cancer cells feeble until cell death occurs. They further stated that acetogenin in soursop also has the propensity to inhibit the NADH: ubiquinone oxidoreductase (Complex I) in the mitochondrial electron transport system and ubiquinone linked with NADH oxidase in tumor cell plasma membranes, which cause the process of programmed cell death (apoptosis).

## 5 Conclusion

The results of this experimentation revealed that different combinations of plant extracts yielded different biological responses. Some combinations exhibited antagonistic effects, while others demonstrated synergistic interactions. This concept of synergism in polyherbal formulations has been a subject of interest in the field, with reports suggesting that combining multiple herbs in precise ratios can enhance the medicinal impact and reduce toxicity. This contrasts with the idea that individual plant extracts may not possess sufficient pharmacological activity on their own. Notably, the Design Expert Software predicted that soursop ethanolic leaf extract, when used in high proportion, could yield the best results, including a strong DPPH radical scavenging effect and high TPC and TFC levels. Soursop’s unique phytochemical composition, characterized by compounds like polyphenols, flavonoids, and acetogenins has been linked to its potent radical-scavenging abilities. Furthermore, the study employed cell-based assays to assess the cytotoxic effects of these plant extracts on cancer cells. The results indicated varying degrees of cytotoxicity, with soursop leaf extract standing out as highly effective, demonstrating a low IC_50_ value, suggesting its potential as a potent anticancer agent. However, caution is necessary when using high concentrations of soursop extract due to its potential cytotoxicity to normal cells. Additionally, the study explored apoptosis induction by soursop leaf ethanolic extract, revealing its ability to trigger programmed cell death.

## Data Availability

The original contributions presented in the study are included in the article/Supplementary Material, further inquiries can be directed to the corresponding authors.
