# Supplementary material for: Anti-proliferative effects of a polyherbal formulation on HL-60, HCT-116, and HeLa cell lines: a docking simulation and response surface design-aided study
Source: Front Chem. 2025 Feb 13;13:1487887. doi: 10.3389/fchem.2025.1487887 (PMC11865080; doi:10.3389/fchem.2025.1487887)
Supplement: Supplementary file 1 [file Table1.docx]

**Supplementary File 1: List of compounds used in this study for molecular docking simulation**

| **S/No** | **PubChem ID** | **Bioactive compounds** |
| --- | --- | --- |
|  | 91535337 | 5,3'-Dihydroxy-6,7,8,4'-tetramethoxyflavanone |
|  | 442793 | [6]-Gingerol |
|  | 72512 | 9-Methoxyellipticine |
|  | 5280442 | Acacetin or Linarigenin |
|  | 393472 | Acetogenins (total annonaceous acetogenins) |
|  | 5280896 | Abscisic acid |
|  | 72965 | Ailanthone |
|  | 65036 | Allicin |
|  | 5971 | Allyl isothiocyanate |
|  | 11503749 | Amooranin |
|  | 656516 | Amygdalin |
|  | 167551 | Anacardic acid |
|  | 5318517 | Andrographolide |
|  | No PubChem ID. 000100* Drawn | Annocatacin A |
|  | No PubChem ID. 000200* Drawn | Annocatacin B |
|  | 10054251 | Annocatalin |
|  | 10054746 | Annohexocin |
|  | 157682 | Annomurine A |
|  | 354398 | Annonacin |
|  | 44259709 | Annulatin |
|  | 637563 | Anethole |
|  | 5280443 | Apigenin |
|  | 9812534 | Aplidine |
|  | 10698768 | Arianacin |
|  | 558221 | (R)-Ar-Turmerone |
|  | 5320351 | Artemetin |
|  | 68827 | Artemisinin |
|  | 6917864 | Artesunate |
|  | 399491 | Artocarpesin |
|  | 54670067 | Ascorbic acid |
|  | 44583916 | Asterosaponin-1 |
|  | 5282102 | Astragalin |
|  | 6710748 | Avocatin B |
|  | 5280682 | Ayanin |
|  | 5281303 | Azadirachtin |
|  | 5281604 | Azaleatin |
|  | 12308714 | Azadiradione |
|  | 64982 | Baicalin |
|  | 10337211 | Bavachinin |
|  | 6456014 | Belotecan |
|  | 21600402 | Benzyl glucosinolate |
|  | 72326 | Betulin |
|  | 275182 | Berbamine |
|  | 2353 | Berberine |
|  | 2355 | Bergapten |
|  | 64971 | Betulinic acid |
|  | 3080597 | Bigelovin |
|  | 5280373 | Biochanin A |
|  | 3778105 | β-Boswellic acid acetate |
|  | 6473739 | Bryostatin-1 |
|  | 5281304 | Bruceantin |
|  | 11124994 | Bullatacin |
|  | 11101102 | Burseran |
|  | 264 | Butyric acid |
|  | 689043 | Caffeic acid |
|  | 5281787 | Caffeic acid phenethyl ester |
|  | 2519 | Caffeine |
|  | 5280453 | Calcitriol |
|  | 5280448 | Calycosin |
|  | 6616 | Camphene |
|  | 2537 | Camphor |
|  | 24360 | Camptothecin |
|  | 5944 | Cantharidin |
|  | 10321 | Capillin |
|  | 1548943 | Capsaicin |
|  | 5315263 | Casticin |
|  | 9064 | Catechin (Cianidanol) |
|  | 65126 | Carnosic acid |
|  | 6419725 | α-Carotene |
|  | 5281515 | β-Caryophyllene |
|  | 7439 | Carvone |
|  | 122724 | Celastrol |
|  | 637760 | Chalcone |
|  | 1794427 | Chlorogenic acid |
|  | 5281607 | Chrysin |
|  | 5280666 | Chrysoeriol |
|  | 637511 | Cinnamaldehyde |
|  | 444539 | Cinnamic acid |
|  | 638011 | Citral |
|  | 311 | Citric acid |
|  | 4485134 | Cleistanthin |
|  | 6167 | Colchicine |
|  | 9895264 | Combretastatin |
|  | 12303802 | Combretol |
|  | 5281233 | Crocin |
|  | 5281235 | β-Cryptoxanthin |
|  | 326 | Cuminaldehyde (4-Isopropylbenzaldehyde) |
|  | 167812 | Curcumenol |
|  | 442360 | α -Curcumene |
|  | 969516 | Curcumin |
|  | 6441391 | Curdione (Germacr-1(10)-ene-5,8-dione) |
|  | 5281232 | Crocetin |
|  | 15224382 | Cycloartocarpesin |
|  | 442972 | Cyclopamine |
|  | 119093 | Cynaropicrin |
|  | 128861 | Cyanidin |
|  | 5281708 | Daidzein |
|  | 68245 | Delphinidin |
|  | 73440 | Dehydroleucodin |
|  | 220401 | Demecolcine |
|  | 16590 | Diallyl disulphide |
|  | 11617 | Diallyl sulphide |
|  | 12232 | Dimethyl disulphide |
|  | 5281612 | Diosmetin |
|  | 5281613 | Diosmin |
|  | 8118 | Dipropyl sulphide |
|  | 5281855 | Ellagic acid |
|  | 3213 | Ellipticine |
|  | 42723 | Elliptinium ( derivative of the alkaloid ellipticine) |
|  | 3220 | Emodin |
|  | 65064 | Epigallocatechin gallate |
|  | 105111 | Epipodophyllotoxin |
|  | 6476031 | Escin IB |
|  | 5281417 | Esculin |
|  | 259331 | Estramustine |
|  | 36462 | Etoposide |
|  | 3314 | Eugenol |
|  | 5317291 | Eupatolitin |
|  | 44259636 | Europetin |
|  | 445858 | Ferulic acid |
|  | 5281614 | Fisetin |
|  | 5356121 | Flavokawin B |
|  | 5287969 | Flavopiridol (Alvocidib) |
|  | 92023653 | Fucoidan |
|  | 5281239 | Fucoxanthin |
|  | 400072 | Galangal acetate |
|  | 96539 | Gardenin B |
|  | 370 | Gallic acid |
|  | 12004512 | Gedunin |
|  | 5280961 | Genistein |
|  | 5281617 | Genkwanin |
|  | 637566 | Geraniol |
|  | 101697178 | Gigantetrocin |
|  | 9909368 | Ginkgolide-A |
|  | 65243 | Ginkgolide-B |
|  | 9867869 | Ginkgolide-C |
|  | 24721483 | Ginkgolide-J |
|  | 9918693 | Ginsenoside Rg3 |
|  | 124052 | Glabridin |
|  | 5464032 | Gluconasturtin |
|  | 9548634 | Glucoraphanin |
|  | 656498 | Glucotropaeolin |
|  | 5317750 | Glycitein |
|  | 14982 | Glycyrrhizic acid |
|  | 44593503 | Goniothalamicin |
|  | 3503 | Gossypol |
|  | 400772 | Halofuginone |
|  | 276389 | Harringtonine, |
|  | 23205 | Helenalin |
|  | 72281 | Hesperetin |
|  | 10082188 | Hispolon |
|  | 73635 | Homoeriodictyol |
|  | 285033 | Homoharringtonine (Omacetaxine mepesuccinate) |
|  | 72303 | Honokiol |
|  | 5281520 | α -Humulene |
|  | 3663 | Hypericin |
|  | 13785311 | Hypoxoside |
|  | 10614148 | Icariside D_2_ |
|  | 10212 | Imperatorin |
|  | 6096870 | Indicaxanthin |
|  | 10177 | Indirubin |
|  | 3712 | Indole-3-carbinol (I3C) |
|  | 15560333 | Intermedeol |
|  | 638014 | β-Ionone |
|  | 5464170 | Irigenin |
|  | 5281255 | Isobavachalcone |
|  | 390361 | Isochamaejasmin |
|  | 638278 | Isoliquiritigenin |
|  | 68079 | Isopimpinellin |
|  | 5281654 | Isorhamnetin |
|  | 162350 | Isovitexin |
|  | 5281166 | Jasmonic acid |
|  | 10326193 | Javoricin |
|  | 102316418 | Jatrophane IV |
|  | 442882 | Justicidin B |
|  | 5280863 | Kaempferol |
|  | 100633 | Karanjin |
|  | 20345 | Kinetin riboside |
|  | 3885 | β-Lapachone |
|  | 102401707 | Laricitin |
|  | 5318998 | Licochalcone A |
|  | 5280450 | α -Linoleic acid |
|  | 5280445 | Luteolin |
|  | 446925 | Lycopene |
|  | 72300 | Magnolol |
|  | 159287 | Malvidin |
|  | 5281650 | α -Mangostin |
|  | 25147451 | Mannan |
|  | 160490 | Matteucinol |
|  | 91466 | Matrine |
|  | 5281828 | Maytansine |
|  | 5281929 | Methyl Jasmonate |
|  | 24832075 | Mezerein |
|  | 169581 | Miconidine |
|  | 9415 | Monocrotaline |
|  | 160679561 | Montamine |
|  | 3035657 | Muricatacin |
|  | 44560014 | Muricatetrocin |
|  | 133072 | Muricatocin B |
|  | 11124830 | Muricin A |
|  | 31253 | β-Myrcene |
|  | 5281672 | Myricetin |
|  | 4276 | Myristicin |
|  | 932 | Naringenin |
|  | 442428 | Naringin |
|  | 442439 | Neohesperidin |
|  | 5317284 | Nepetin |
|  | 108058 | Nimbin |
|  | 12313376 | Nimbolide |
|  | 72344 | Nobiletin |
|  |  | Noscapine |
|  | 332427 | Lariciresinol |
|  | 5318998 | Licochalcone A |
|  | 73636 | Liriodendrin |
|  | 114829 | Liquiritigenin |
|  | 259846 | Lupeol |
|  | 100771 | Obovatol |
|  | 5320287 | Ombuin |
|  | 5321010 | Oridonin |
|  | 5320315 | Oroxylin A |
|  | 5281677 | Pachypodol |
|  | 7251185 | Parthenolide |
|  | 637542 | P-Coumaric acid (4-Hydroxycinnamic acid) |
|  | 36314 | Paclitaxel |
|  | 6918506 | Peloruside A |
|  | 6440892 | Picroside I |
|  | 638024 | Piperine |
|  | 637858 | Piperlongumine |
|  | 10995620 | Pervilleine |
|  | 101844812 | Phanginin D |
|  | 219100 | Phenoxodiol (Idronoxil) |
|  | 442070 | Phorbol |
|  | 11558520 | Phoyunbene B |
|  | 130796 | Picrocrocin |
|  | 91508 | α -Pinene |
|  | 73399 | Pinoresinol |
|  | 162859 | Platycodin |
|  | 10205 | Plumbagin |
|  | 10607 | Podophyllotoxin |
|  | 72960700 | Polyphyllin |
|  | 4871 | Pomiferin |
|  | 5281803 | Pratensein |
|  | 135455579 | Prodigiosin |
|  | 9920281 | Protopanaxadiol |
|  | 5281804 | Prunetin |
|  | 5353911 | Psi-tectorigenin |
|  | 6199 | Psoralen |
|  | 101324849 | Pterokaurane |
|  | 5281727 | Pterostilbene |
|  | 5280343 | Quercetin |
|  | 445154 | Resveratrol |
|  | 5352005 | Retusin |
|  | 5281691 | Rhamnetin |
|  | 10168 | Rhein |
|  | 13422573 | Rohitukine |
|  | 160355 | (R)-Roscovitine (Seliciclib) |
|  | 5281792 | Rosmarinic |
|  | 5280805 | Rutin |
|  | 61041 | Safranal |
|  | 3085092 | Salinomycin |
|  | 73571 | Sakuranetin |
|  | 5280460 | Scopoletin |
|  | 5281697 | Scutellarein |
|  | 9917980 | Secoisolariciresinol diglucoside |
|  | 442872 | Securinine |
|  | 479503 | Shikonin |
|  | 145659 | Sinensetin |
|  | 23682211 | Sinigrin (Allyl glucosinolate) |
|  | 11787114 | Silvesterol |
|  | 5213 | Silymarin |
|  | 11376469 | Solamin |
|  | 262500 | Solanine |
|  | 509245 | Sophoraflavanone B |
|  | 1268276 | Sterubin (7-O-Methyleriodictyol) |
|  | 5280794 | Stigmasterol |
|  | 222284 | β-sitosterol |
|  | 5350 | Sulforaphane |
|  | 51683 | Swainsonine |
|  | 7172 | Synephrine |
|  | 5281699 | Tamarixetin |
|  | 68077 | Tangeretin |
|  | 875 | Tartaric acid |
|  | 215159 | Taspine |
|  | 9548828 | Taxane |
|  | 452548 | Teniposide |
|  | 476861 | Terameprocol |
|  | 443162 | α -Terpineol |
|  | 73078 | Tetrandrine |
|  | 10281 | Thymoquinone |
|  | 122206355 | Tinosporin A |
|  | 65576 | Tomatidine |
|  | 60700 | Topotecan |
|  | 5281701 | Tricetin |
|  | 5570 | Trignolline |
|  | 51346132 | Tubeimoside-1 |
|  | 5281426 | Umbelliferone |
|  | 135515151 | Undecylprodigiosin |
|  | 64945 | Ursolic acid |
|  | 8468 | Vanillic acid |
|  | 5281800 | Verbascoside (Acteoside) |
|  | 442664 | Vicenin-2 |
|  | 13342 | Vinblastine |
|  | 5978 | Vincristine |
|  | 40839 | Vindesine |
|  | 6918295 | Vinflunine (Javlor) |
|  | 5311497 | Vinorelbine |
|  | 5280441 | Vitexin |
|  | 74947464 | Vitisin B |
|  | 479756 | Wikstromol ((+)-Nortrachelogenin) |
|  | 53477765 | Withanolide |
|  | 5281703 | Wogonin |
|  | 629965 | Zapotin |
|  | 92776 | Zingiberene |
