# Supplementary material for: Anti-proliferative effects of a polyherbal formulation on HL-60, HCT-116, and HeLa cell lines: a docking simulation and response surface design-aided study
Source: Front Chem. 2025 Feb 13;13:1487887. doi: 10.3389/fchem.2025.1487887 (PMC11865080; doi:10.3389/fchem.2025.1487887)
Supplement: Supplementary file 2 [file Table2.docx]

**Supplementary File 2: List of standard inhibitors used in this study with their respective PubChem IDs**

| **S/No** | **Name of Inhibitor/drug** | **PubChem ID** | **Target** |
| --- | --- | --- | --- |
|  | Sorafenib | 216239 | FLT3 |
|  | Gilteritinib | 49803313 | FLT3 |
|  | Ivosidenib | 71657455 | IDH1 |
|  | Enasidenib | 89683805 | IDH2 |
|  | Ipatasertib | 24788740 | AKT1 |
|  | A-674563 | 11314340 | AKT1 |
|  | Wortmannin | 312145 | PI3K |
|  | LY294002 | 3973 | PI3K |
|  | Rapamycin | 5284616 | mTOR |
|  | Temsirolimus | 6918289 | mTOR |
|  | T4K-733 | 24963252 | MEK1 |
|  | Selumetinib | 10127622 | MEK1 |
|  | Trametinib | 11707110 | MEK1 |
|  | Cobimetinib | 16222096 | MEK1 |
|  | Fedratinib | 16722836 | JAK2 |
|  | Gandotinib | 46213929 | JAK2 |
|  | Baricitinib | 44205240 | JAK2 |
|  | AC-4-130 | 154701370 | STAT5 |
|  | BP-1-108 | 56663448 | STAT5 |
|  | Daurismo (Glasdegib) | 25166913 | SMO |
|  | Sonidegib | 24775005 | SMO |
|  | IWP -4 | 2155264 | β-Catenin |
|  | Cardionogen 1 | 663145 | β-Catenin |
|  | Dithiocarbamate | 3037131 | NF-kB |
|  | Disulfiram | 3117 | NF-kB |
|  | Olmesartan | 158781 | NF-kB |
|  | Venetoclax (ABT-199) | 49846579 | Bcl-2 |
|  | Abt-737 (ABT-737) | 11228183 | Bcl-2, Bcl-xL and Bcl-w |
|  | Navitoclax (ABT-263) | 24978538 | Bcl-2 |
|  | Obatoclax | 11404337 | Inhibitor of the Bcl-2 family of proteins |
|  | Guadecitabine | 135564655 | DMNT1 |
|  | Azacitidine | 9444 | DMNT1 |
|  | Vorinostat | Vorinostat | HDAC |
|  | Pracinostat | 49855250 | HDAC |
|  | 2,4-pyridinedicarboxylic acid | 10365 | HDM |
|  | Disulfiram | 3117 | HDM |
|  | AS8351 | 135400486 | HDM |
|  | LTK-14 | 25226432 | HAT |
|  | C646 | 1285941 | HAT |
|  | Garcinol | 5281560 | HAT |
|  | Geldanamycin | 5288382 | Hsp90 |
|  | Radicicol | 6323491 | Hsp90 |
|  | Tanespimycin | 6505803 | Hsp90 |
|  | Luminespib | 135539077 | Hsp90 |
|  | MKT-077 | 6912334 | Hsp70 |
|  | VER-155008 | 25195348 | Hsp70 |
|  | Apoptozole | 24894064 | Hsp70 |
|  | Indazole | 9221 | CYP2E1 |
|  | 4-Methylpyrazole | 3406 | CYP2E1 |
|  | Disulfiram | 3117 | CYP2E1 |
|  | Idarubicin | 42890 | Topoisomerase II poison (Prevents DNA unwinding) |
|  | Daunorubicin | 30323 | Interacts with DNA by intercalation and inhibition of macromolecular biosynthesis |
